# Effect of Pufa Substrates on Fatty Acid Profile of *Bifidobacterium breve* Ncimb 702258 and CLA/CLNA Production in Commercial Semi-Skimmed Milk

**DOI:** 10.1038/s41598-018-33970-2

**Published:** 2018-10-22

**Authors:** Ana Luiza Fontes, Lígia Pimentel, Luis Miguel Rodríguez-Alcalá, Ana Gomes

**Affiliations:** 1 Universidade Católica Portuguesa, CBQF - Centro de Biotecnologia e Química Fina – Laboratório Associado, Escola Superior de Biotecnologia, Porto, 4200-374 Portugal; 2 CINTESIS – Centro de Investigação em Tecnologias e Sistemas de Informação em Saúde, Faculdade de Medicina da Universidade do Porto, Porto, 4200-450 Portugal; 3 QOPNA – Unidade de Investigação de Química Orgânica, Produtos Naturais e Agroalimentares, Universidade de Aveiro, Campus Universitário de Santiago, Aveiro, 3810-193 Portugal; 4 Centro de Investigación en Recursos Naturales y Sustentabilidad (CIRENYS), Universidad Bernardo O’Higgins, Santiago, de Chile Chile

## Abstract

Current research on lipids is highlighting their relevant role in metabolic/signaling pathways. Conjugated fatty acids (CFA), namely isomers of linoleic and linolenic acid (i.e. CLA and CLNA, respectively) can positively modulate inflammation processes and energy metabolism, promoting anti-carcinogenic and antioxidant effects, improved lipid profiles and insulin resistance, among others. Bioactive doses have been indicated to be above 1 g/d, yet these cannot be achieved through a moderate intake (i.e. 1–2 servings) of natural sources, and certain CLA-containing products have limited commercial availability. Such handicaps have fueled research interest in finding alternative fortification strategies. In recent years, screening of dairy products for CFA-producing bacteria has attracted much attention and has led to the identification of some promising strains, including *Bifidobacterium breve* NCIMB 702258. This strain has shown interesting producing capabilities in model systems as well as positive modulation of lipid metabolism activities in animal studies. Accordingly, the aim of this research work was to assay *B. breve* NCIMB 702258 in semi-skimmed milk to produce a probiotic fermented dairy product enriched in bioactive CLA and CLNA. The effect of substrates (LA, α-LNA and γ-LNA) on growth performance and membrane fatty acids profile was also studied, as these potential modifications have been associated to stress response. When tested in cys-MRS culture medium, LA, α-LNA and γ-LNA impaired the fatty acid synthesis by *B. breve* since membrane concentrations for stearic and oleic acids decreased. Variations in the C18:1 c11 and lactobacillic acid concentrations, may suggest that these substrates are also affecting the membrane fluidity. *Bifidobacterium breve* CFA production capacity was first assessed in cys-MRS with LA, α-LNA, γ-LNA or all substrates together at 0.5 mg/mL each. This strain did not produce CFA from γ-LNA, but converted 31.12% of LA and 68.20% of α-LNA into CLA and CLNA, respectively, after incubation for 24 h at 37 °C. In a second phase, *B. breve* was inoculated in a commercial semi-skimmed milk with LA, α-LNA or both at 0.5 mg/mL each. *Bifidobacterium breve* revealed a limited capacity to synthesize CLA isomers, but was able to produce 0.062–0.115 mg/mL CLNA after 24 h at 37 °C. However, organoleptic problems were reported which need to be addressed in future studies. These results show that although CFA were produced at too low concentrations to be able to achieve solely the bioactive dose in one daily portion size, fermented dairy products are a suitable vector to deliver *B. breve* NCIMB 702258.

## Introduction

Among the family of bioactive conjugated fatty acids (CFA), those isomers from linoleic acid (CLA) and more recently those from linolenic acid (CLNA) have attracted much attention due to their potential as anti-carcinogenic, anti-inflammatory, immunomodulatory, anti-obesity and antioxidant compounds^1–3^. Accordingly, these fatty acids (FA) are considered promising new bioactive ingredients and several research works are focused on finding high content sources.

CLA isomers are naturally present in meat (1.2–17 mg CLA/g fat) and ruminants milk (0.55–9.2 mg CLA/g fat)^4^. CLNA isomers are found at lower amounts in these foods (1–3 mg CLNA/g fat in milk and 9–27 mg CLNA/g fat in meat)^5^ but are principally found in vegetable oils (10–700 mg CLNA/g oil), such as tung oil, pot marigold and pomegranate seed oils^6^. To benefit from these CFA bioactive properties, high effective doses have been recommended, namely 3–6 g/day for CLA for a 70 kg person^7^ and 2–3 g/day for CLNA^8^, which hampers the current use of such natural sources in human consumption if nutritional recommended daily doses are to be maintained. The lower dose required for CLNA may be associated to some results where these CFA are metabolized into CLA in rats^9,10^. Nevertheless, other mechanistic effects through peroxisome proliferator-activated receptor alpha (PPARα) activation in adipocytes^11^ cannot be discarded. These amounts are not feasible through CLA/CLNA natural sources, not to mention other limitations in terms of commercial availability and safety.

Interestingly, both CLA and CLNA isomers are intermediates of rumen biohydrogenation of dietary linoleic acid (LA) and linolenic acid (LNA) to stearic acid (C18) mainly by *Butyrivibrio fribrisolvens*, but also by other bacteria^12,13^. There is also an enzymatic synthesis of CLA by Δ9-desaturase in the mammary gland of lactating cows, through conversion of trans-vaccenic acid (C18:1 t11; TVA)^14^. However, it has been reported that ruminal bacteria are not the only ones capable of producing CLA and CLNA. Other species isolated from dairy products and human gastrointestinal tract have shown a similar capacity, namely, strains of lactobacilli, propionibacteria and bifidobacteria^6,15–17^. Over the last years, the latter have proven to be promising producers: Raimondi *et al*.^18^ found that 41.8–88.1% LA (0.5 mg/mL) added to culture medium was converted to CLA isomers. Furthermore, when tested substrate was α-LNA (0.37 mg/mL), bifidobacteria from neonatal gastrointestinal tract, namely *B. breve* and *B. pseudocatenulatum* species, revealed conversion rates into CLNA between 46.3% and 90.5%^19^. Such differences between strains or between species may be related with the different linoleate isomerase activity associated^6^.

Fatty acid synthesis by microorganisms plays an important role in their adaptation to stress and proliferation such that inhibition of this pathway is currently been assayed as alternative solutions to control pathogenic strains^20,21^. Interestingly, unsaturated fatty acids and mainly LA, have been proven to alter the activity of bacterial enzymes involved in the elongation limiting-steps^22^ in agreement with investigations suggesting that CFA production is a detoxifying mechanism^23,24^. Accordingly, this research work raises the hypothesis that inhibition by these FA is associated with alteration of membrane lipids profile, possibly changes in the levels of palmitic or stearic acids.

Dairy milk appears as a suitable matrix for the microbial production of CFA by such lactic acid bacteria with interesting perspectives for further commercial applications. Numerous fermented dairy products reveal higher contents of CLA than their non-fermented counterparts. For example, in organic milk, *B. animalis* subsp. *lactis* strains improved CLA concentration by 65% up to 1.85 g/100 g when compared to control non-fermented milk (1.15 g/100 g)^25^. Moreover, fermentation of buffalo milk with *B. bifidum* CRL1399 and sunflower oil (0.2 mg/mL of LA) led to the production of a fresh cheese with higher CLA content (6.6 mg/g of fat) than raw milk alone (4.9 mg/g of fat)^26^. More recently, Villar-Tajadura *et al*.^27^ have succeeded in elaborating a skim-milk containing 0.48 mg CLNA/mL by assaying several bifidobacteria strains isolated from breastmilk.

Furthermore, this microbial genus has shown surprising health properties. Highly efficient CLA/CLNA producing *Bifidobacterium* strains, namely *B. breve* NCIMB 702258, increased *in vivo* the concentration of rumenic acid (RA; C18:2 c9t11 CLA) in murine and pigs’ livers^28^ as well as DHA and EPA in mice’s adipose tissue and brain^29^. Furthermore, this strain has recently revealed excellent capabilities in modulating host’s lipid metabolism^30^ by increasing adipose tissue ß-oxidation and lowering liver fat uptake. Nevertheless, despite its capacities to remodel and modulate both body lipid composition and metabolism, to the best of our knowledge there is a lack of studies focused on elaborating functional products with this strain. Such studies represent an interesting approach in the production of future novel functional products to control the rocketing increment of obesity. Accordingly, the aim of this research work was to investigate the possibilities of using lipid-regulating *B. breve* NCIMB 702258 in the production of a CLA-enriched fermented dairy product using commercially available milk as a first step in the production of commercial functional food products.

## Results

### Tolerance to substrates

With regard to LA inhibitory effect on bacterial growth, some authors^31,32^ have suggested that LA inhibition is not species- but strain dependent, since among a considerable amount of bifidobacteria and lactic acid bacteria strains only a few revealed inhibited growth and fewer were completely inhibited by LA. Moreover, Coakley *et al*.^33^ reported that all five *L. reuteri* strains tested were able to grow with LA up to 1 mg/mL and, inclusively, three of them tolerated up to 3 mg/mL. On the other hand, other researchers, when assessing a *L. lactis* strain observed that it could not grow at LA concentrations above 0.5 mg/mL^34^.

**Figure 1:**
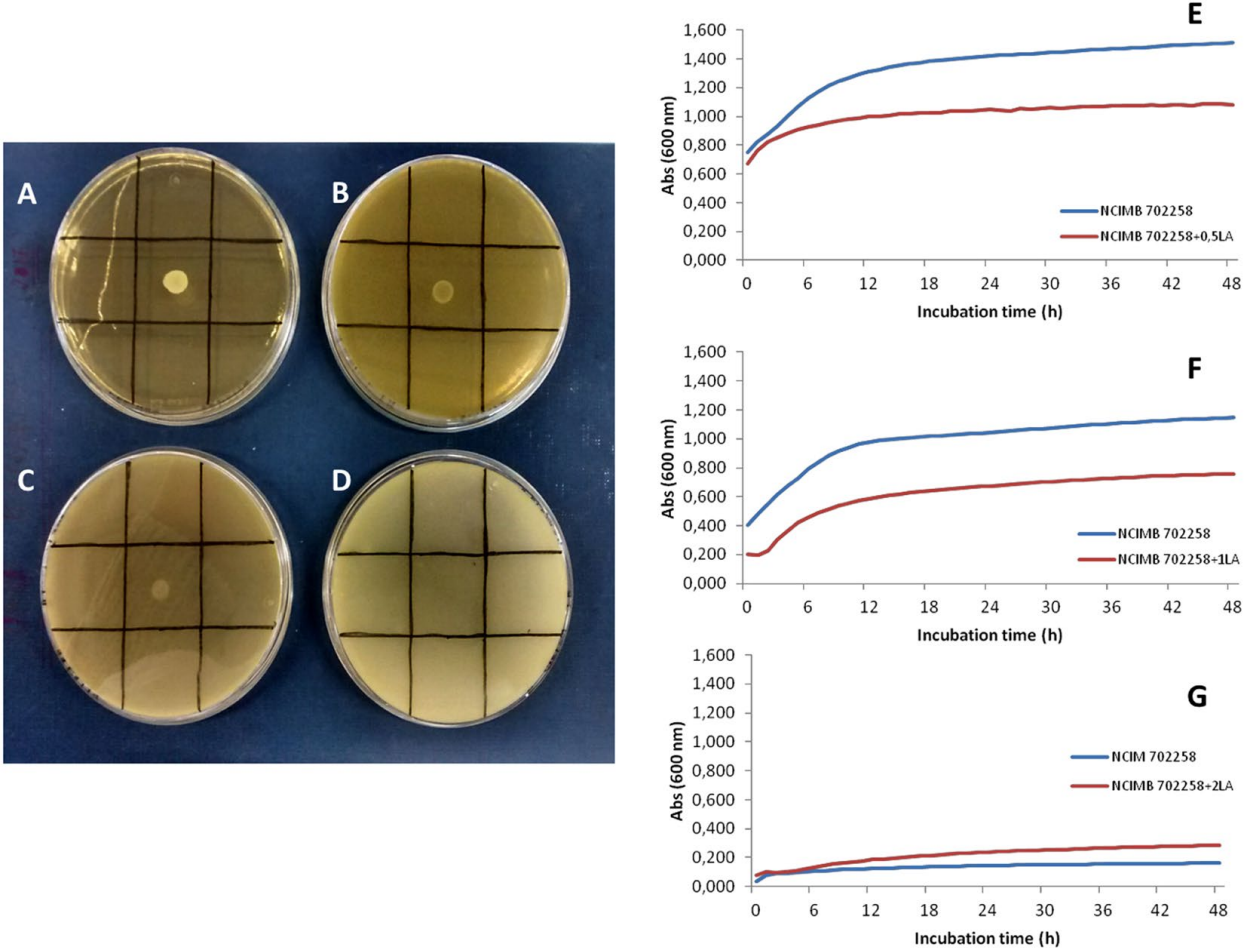
Substrate tolerance of *B. breve* NCIMB 702258 in cys-MRS agar plates containing 0 (**A**), 1 (**B**), 2 (**C**) and 5 mg/mL (**D**) of LA and growth curves in the presence of 0.5 (**E**), 1 (**F**) and 2 mg LA/mL (**G**) after 48 h incubation at 37 °C, under anaerobic conditions. Optical density was recorded at 600 nm. Results are expressed as mean and standard deviation (n = 3).

When assaying *B. breve* NCIMB 702258 in agar plates containing 0.1–0.5% LA (Fig. 1 A–D), it was observed that the higher the initial LA concentration added the lower the strain’s growth capacity, which was completely inhibited at 0.5% LA (Fig. 1D). However, although this bacterium seemed to tolerate up to 0.1% LA (i.e. 1 mg/mL), negative effects could not be excluded. Thus, in a further experiment *B. breve* NCIMB 702258 was initially grown on 0, 0.1 and 0.2% LA containing agar plates (i.e. LA concentrations enabling growth capacity) as a pre-adaptation step and growth capacity was subsequently tested in medium broth without and with 0.05, 0.1 and 0.2% LA by OD_600_ measurements in a microplate reader (Fig. 1E–G); note that 0.05% was introduced in this second step to increase growth possibilities.

Figure 1E (blue line) depicts the typical growth curve for this *Bifidobacterium* strain under the assayed conditions. On the other hand, obtained results clearly point out that 0.2% (2 mg LA/mL) in agar plates produced sublethal injury to *B. breve* NCIMB 702258 since it was unable to further replicate its potential growth capacity in the absence of this FA (Fig. 1G, blue line). When comparing Figure 1E (0.5 mg LA/mL) vs 1F (1 mg LA/mL), absorbance values at 600 nm were lower in the 1 mg/mL experiment in both control (pre-adaptation in 0.1% LA containing plates; blue line) and test sample (growth in 0.1% LA containing medium broth upon pre-adaptation step; red line). Moreover, for this latter situation a 3-h delay in the exponential growth phase was observed and *B. breve* NCIMB 702258 also seemed to reach the stationary phase earlier (Fig. 1F; red line vs blue line of control). Accordingly, the following experiments assaying CFA production in cys-MRS medium and milk were carried out at the lowest concentration tested of 0.5 mg/mL LA.

During this tolerance test, LA was assayed as the representative FA of the 18-carbon chain PUFA family. However, previous works have reported that LNA is able to inhibit *Butyrivibrio fibrisolvens* growth to a greater extent than LA^35^. Furthermore, Hennessey *et al*. demonstrated that *B. breve* NCIMB 702258 was able to grow in the presence of LA, α-LNA, γ-LNA, however specific inhibitory effects by these substrates were not reported^36^.

**Figure 2:**
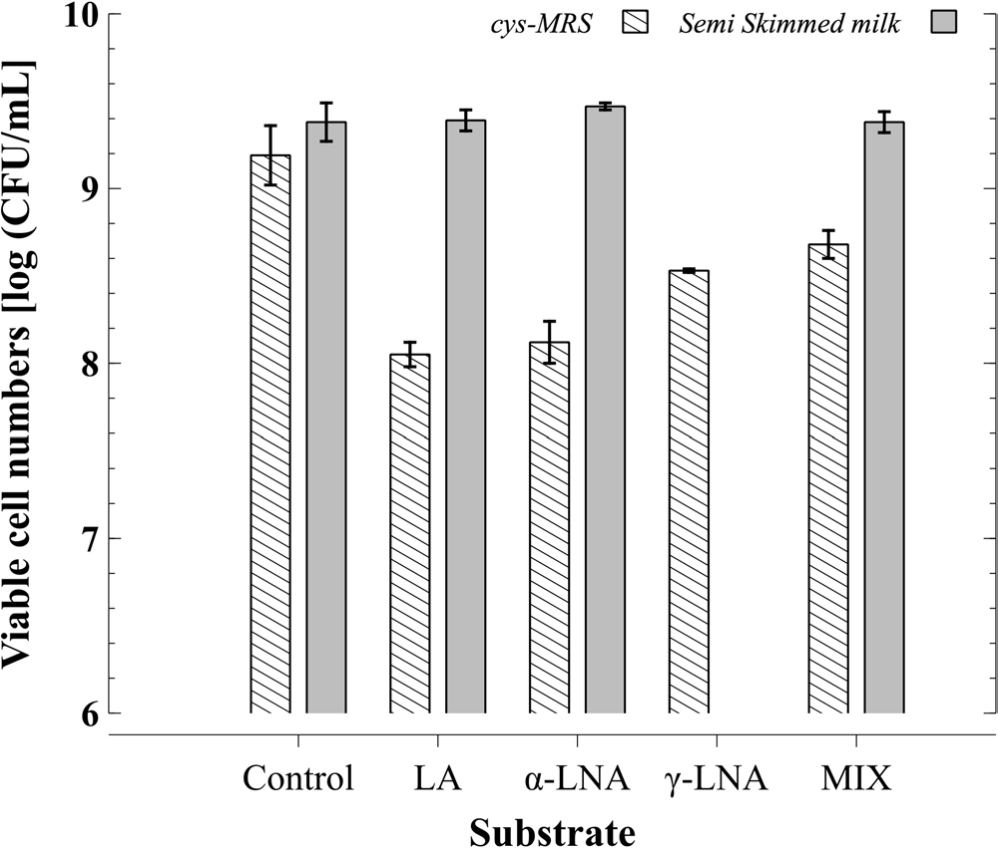
Viable cell numbers of *B. breve* NCIMB 702258 [log (CFU/mL)] after 24 h incubation in cys-MRS culture medium or in commercial semi-skimmed UHT milk added with different unsaturated fatty acids. LA, α-LNA, γ-LNA or the three FA mixture (MIX) were in a final concentration of 0.5 mg/mL for each unsaturated fatty acid. γ-LNA was not assayed either alone or in MIX in semi-skimmed milk since previous assays showed no CFA production.

According to the obtained data, viable cell numbers of *B. breve* NCIMB 702258 upon 24 h of incubation in cys-MRS added with different precursor FA (Fig. 2) reached levels between 8–9 log cfu/mL. These values where not significantly different when compared to those obtained in control (>9 log cfu/mL). Interestingly, the following trend was observed: LA < α-LNA < γ-LNA < MIX (p > 0.05).

### CFA production in culture medium

**Figure 3:**
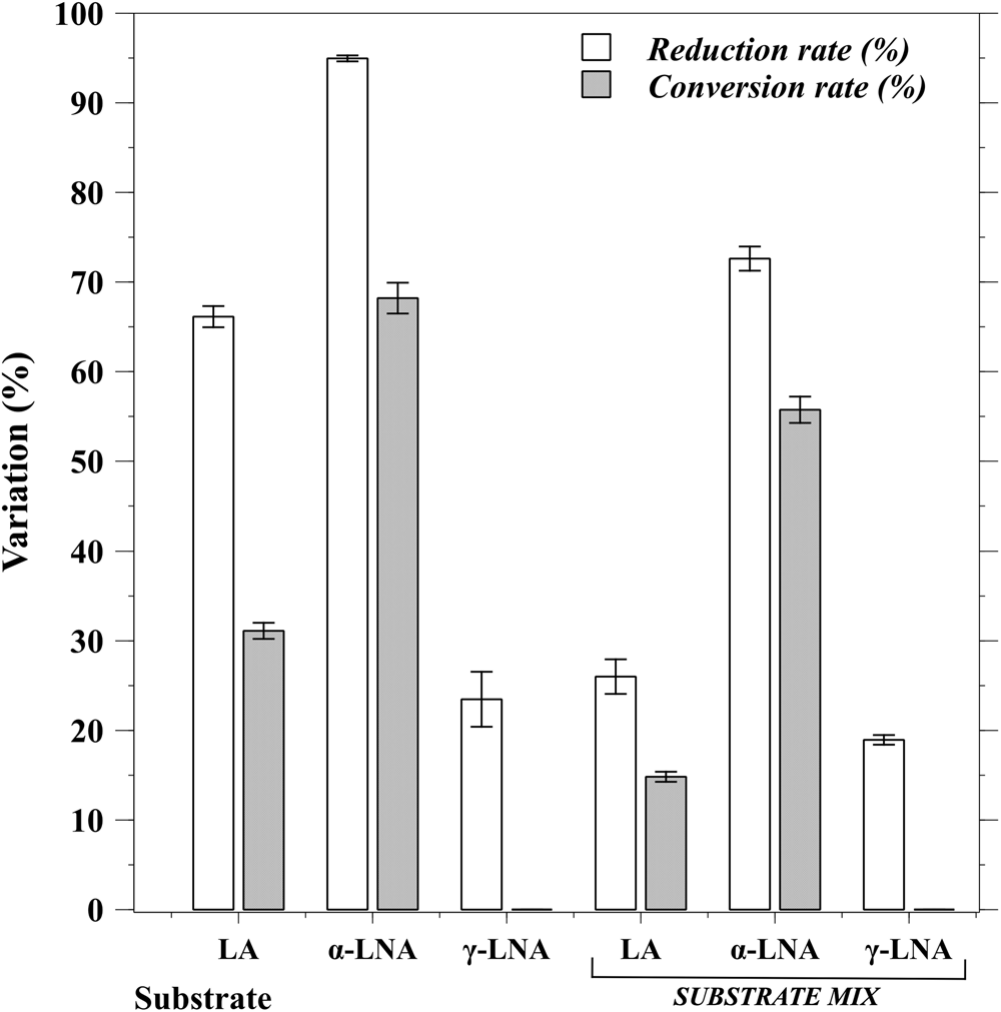
Substrate percentage reduction of different polyunsaturated fatty acids and conversion into corresponding conjugated fatty acids (%) by *B. breve* NCIMB 702258 in cys-MRS culture medium after 24 h incubation at 37 °C under anaerobic conditions.

In what concerns CFA production capacity in cys-MRS culture medium supplemented with 0.5 mg/mL FA, results are illustrated in Figure 3. *Bifidobacterium breve* NCIMB 702258 was able to transform 31.1% LA into CLA isomers and 68.2% of α-LNA into CLNA isomers. However, this strain was not able to use γ-LNA for CFA synthesis, even though its concentration in the culture medium was reduced in 23.5% upon 24 h of incubation.

**Figure 4:**
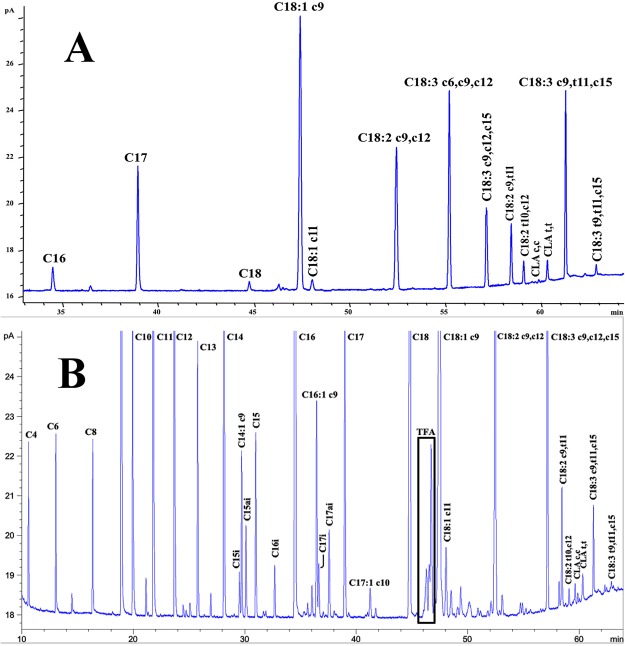
Fatty acid composition of cys-MRS culture medium (**A**) and commercial semi-skimmed UHT milk (**B**) assayed for conjugated fatty acid production by *B. breve* NCIMB 702258 using linoleic (LA) and linolenic (α-LNA) acids as precursor substrates. A mixture of LA/α-LNA/γ-LNA in cys-MRS culture medium and LA/α-LNA in semi-skimmed UHT milk were assayed at a concentration of 0.5 mL/mg for each fatty acid.

In the presence of all three FA (LA/α-LNA/γ-LNA, 0.5 mg/mL each), *B. breve* NCIMB 702258 was able to transform 14.8% LA and 55.7% α-LNA into CLA and CLNA, respectively; the main isomers found were C18:2 c9,t11 and C18:3 c9,t11,c15 (Fig. 4A). Accordingly, α-LNA, LA and γ-LNA concentrations were reduced 72.6%, 26.0% and 19.0%, respectively.

Based on the results related to γ-LNA, this FA was not further assayed in commercial semi-skimmed milk.

### Effects of PUFA in the membrane FA composition

To better understand the behavior of this strain in the presence of the different tested substrates (LA, α-LNA, γ-LNA or all 3 substrates together) the FA composition of pellets obtained after 24 h in cys-MRS culture medium was analyzed (Table 1). Nine FA were detected in the control samples (CRL), the main compounds being palmitic (C16) and oleic (C18:1 c9) acids. Neither LA, α-LNA, γ-LNA or CLNA were detected while C18:2 c9,t11, C18:2 t10,c12 and C18:2 CLA t,t registered low concentrations of 0.031 ± 0.010, 0.044 ± 0.006 and 0.057 ± 0.009 µg/mg, respectively. CLA isomers were the main compounds in the PUFA fraction while oleic acid (2.084 ± 0.131 µg/mg) was the main compound among the MUFA.

**Table 1:** Fatty acid composition (µg/mg) of pellets from *B. breve* NCIMB 702258 after 24 h of incubation in cys-MRS culture medium control (CRL) and with 0.5 mg/mL of LA (S1), α-LNA (S2), γ-LNA (S3) and LA + α-LNA + γ-LNA (1:1:1; MX).

Fatty acid*	CRL	S1	S2	S3	MX
C14	0.410 ± 0.022^a^	0.341 ± 0.025^b^	0.232 ± 0.023^c^	0.095 ± 0.012^d^	0.107 ± 0.011^d^
C16	1.958 ± 0.047^b^	1.879 ± 0.140^b^	2.307 ± 0.231^a^	1.529 ± 0.082^c^	1.858 ± 0.186^b^
C18	0.304 0.007^a^	0.151 ± 0.011^d^	0.214 ± 0.021^b^	0.136 ± 0.008^e^	0.188 ± 0.019^c^
C18:1 c9	2.084 ± 0.131^a^	1.974 ± 0.147^a^	1.531 ± 0.153^b^	0.802 ± 0.036^c^	0.858 ± 0.086^c^
C18:1 c11	0.139 ± 0.009^c^	0.403 ± 0.030^a^	0.228 ± 0.023^b^	0.087 ± 0.008^e^	0.109 ± 0.011^d^
C18:2 c9,c12	<LOD^c^	2.179 ± 0.162^a^	<LOD^c^	<LOD^c^	0.784 ± 0.078^b^
cyc 19:0	0.369 ± 0.023^a^	0.167 ± 0.012^b^	0.178 ± 0.018^b^	0.154 ± 0.008^c^	0.116 ± 0.012^d^
C18:3 c6,c9,c12	<LOD^c^	<LOD^c^	<LOD^c^	0.830 ± 0.018^a^	0.637 ± 0.064^b^
C18:3 c9,c12,c15	<LOD^b^	<LOD^b^	0.229 ± 0.023^a^	<LOD^b^	0.236 ± 0.024^a^
C18:2 c9,t11	0.031 ± 0.010^c^	1.774 ± 0.132^a^	<LOD^d^	<LOD^d^	0.229 ± 0.023^b^
C18:2 t10,c12	0.044 ± 0.006^a^	<LOD^b^	<LOD^b^	<LOD^b^	0.048 ± 0.005^a^
C18:2 CLA t,t	0.057 ± 0.009^c^	0.243 ± 0.018^a^	<LOD^d^	<LOD^d^	0.090 ± 0.009^b^
C18:3 c9,t11,c15	<LOD^c^	<LOD^c^	1.790 ± 0.179^a^	<LOD^c^	0.551 ± 0.055^b^
C18:3 t9,t11,c15	<LOD^c^	<LOD^c^	0.249 ± 0.086^a^	<LOD^c^	0.144 ± 0.014^b^
Σ CLA	0.133 ± 0.025^c^	2.017 ± 0.150^a^	<LOD^d^	<LOD^d^	0.367 ± 0.037^b^
Σ CLNA	<LOD^c^	<LOD^c^	2.039 ± 0.204^a^	<LOD^c^	0.695 ± 0.070^b^
Σ SFA	3.041 ± 0.099^a^	2.539 ± 0.189^b^	2.931 ± 0.293^a^	1.914 ± 0.078^d^	2.269 ± 0.227^c^
Σ MUFA	2.223 ± 0.140^a^	2.377 ± 0.177^a^	1.759 ± 0.176^b^	0.888 ± 0.044^c^	0.967 ± 0.097^c^
Σ PUFA	0.133 ± 0.025^e^	4.196 ± 0.312^a^	2.268 ± 0.227^c^	0.830 ± 0.018^d^	2.719 ± 0.272^b^
Σ FA	5.397 ± 0.264^c^	9.112 ± 0.678^a^	6.959 ± 0.696^b^	3.633 ± 0.016^d^	5.955 ± 0.596^c^

^*^Average value ± standard deviation (n = 3).

^a,b,c,d,e^Different superscript letters within a row for significant differences (p < 0.05).

c = *cis* double bound; t = *trans* double bound; cyc = cyclopropane fatty acid; Σ = total; CLA = conjugated linoleic acid; CLNA = conjugated linolenic acid; SFA = saturated fatty acids; MUFA = monounsaturated fatty acids; PUFA = polyunsaturated fatty acids; FA = fatty acids; <LOD = below limit of detection.

The addition of LA (S1), α-LNA (S2), γ-LNA (S3) alone or together (MX) to cys-MRS culture medium modified the overall pellet FA composition, both qualitatively and quantitatively; total SFA were decreased, total MUFA were maintained (S1) or decreased (S2, S3 and MX) and total PUFA were significantly increased, in particular for S1, S2 and MX, in comparison to the CRL (Table 1; p < 0.05). As expected, the CFA profile was also affected, increasing significantly total CLA concentrations in S1 and total CLNA concentrations in S2 in comparison to the control (p < 0.05); total CLA and CLNA were below detection limits in S3 and although increases in total CLA (0.367 ± 0.037 µg/mg) and CLNA (0.695 ± 0.070 µg/mg) were reported for MX these were of lower order of magnitude when compared to S1 and S2.

From a more specific perspective, the addition of LA (S1) to cys-MRS culture medium led to the maintenance of palmitic and oleic acids at similar concentrations to the control, yet myristic (C14), stearic (C18) and lactobacillic acid (cyc C19) decreased (p < 0.05) when compared to CRL, reflected in the lower content of total SFA. Concentration of C18:1 c11 ranged from 0.139 ± 0.009 µg/mg in CRL to 0.403 ± 0.030 µg/mg in S1 samples (p < 0.05). A similar increasing pattern was found for S2 (p < 0.05) while S3 samples showed the lowest concentration (p < 0.05). In what concerns CFA and PUFA concentrations, the addition of LA (S1) led to a pronounced increment in its concentration in the corresponding pellets (2.179 ± 0.162 µg/mg) and total FA (9.112 ± 0.678 µg/mg) (p < 0.05). Concentrations of both RA and C18:2 CLA t,t were also raised, while C18:2 t10, c12 completely disappeared to levels below detection limit. On the other hand, α-LNA accumulation in S2 samples was rather limited (0.229 ± 0.023 µg/mg) unlike CLNA compounds, where both isomers were detected at concentration levels of 1.790 ± 0.179 µg/mg for C18:3 c9,t11,c15 and 0.249 ± 0.086 µg/mg for C18:3 t9,t11,c15.

In the supernatants from cys-MRS added with γ-LNA (S3), neither CLA or CLNA were detected. γ-LNA concentration was also low (0.830 ± 0.018 µg/mg) when compared with LA accumulation in S1 but higher than α-LNA in S2. Total FA content in S3 samples was the lowest registered (3.633 ± 0.016 µg/mg; p < 0.05).

When all three FA substrates were added to the growth-medium (MX), the presence of C18:2 t10,c12 was detected. α-LNA accumulation (0.236 ± 0.024 µg/mg) was similar to that in S2 (p > 0.05) while for γ-LNA (0.637 ± 0.064 µg/mg) and LA (0.784 ± 0.078 µg/mg) concentrations were significantly lower (p < 0.05).

When TMS derivatization was performed, hydroxy FA or other additional compounds were not detected in any of the analyzed pellets.

### CFA production in commercial semi-skimmed milk

The viable cell numbers of *B. breve* NCIMB 702258 achieved upon incubation in commercial semi-skimmed milk (ML) (Figure 2) revealed that ML is a more suitable medium to promote the growth of this *Bifidobacterium* strain than cys-MRS culture medium.

The FA composition of the commercial semi-skimmed milk (ML) used to test CFA producing capabilities of *B. breve* NCIMB 702258 in a dairy matrix is presented in Table 2. In general, the qualitative profile is of a broader nature given the natural richness of milk in short and medium chain FA, besides the unsaturated fatty acids (UFA). Results showed that, as expected, the main FA in milk (ML) were palmitic (C16; 4.263 ± 0.026 mg/mL), oleic (C18:1 c9; 2.761 ± 0.021 mg/mL) and stearic acids (C18; 1.420 ± 0.009 mg/mL). Corresponding concentrations for LA and α-LNA were 0.220 ± 0.001 and 0.054 ± 0.002, respectively. The presence of CLA, mainly RA (0.083 ± 0.003 mg/mL) was also detected, while CLNA isomers (C18:3 c9,t11,c15 and C18:3 t9,t11,c15) were found at very low concentrations.

**Table 2:** Fatty acid composition (mg/mL) of semi-skimmed UHT milk (ML) and semi-skimmed fermented UHT milk prepared with *B. breve* NCIMB 702258 upon 24 h incubation in the absence (FCRL) and presence of either 0.5 mg/mL of LA (FS1), α-LNA (FS2) or both substrates (1:1; FMX).

Fatty acid*	ML	FCRL	FS1	FS2	FMX
C4	0.092 ± 0.001^a^	0.075 ± 0.006^bc^	0.067 ± 0.001^d^	0.071 ± 0.005^cd^	0.079 ± 0.000^b^
C6	0.108 ± 0.001^a^	0.087 ± 0.008^bc^	0.077 ± 0.000^d^	0.083 ± 0.005^c^	0.094 ± 0.000^b^
C8	0.100 ± 0.001^a^	0.084 ± 0.007^bc^	0.072 ± 0.002^d^	0.077 ± 0.006^cd^	0.086 ± 0.001^b^
C10	0.314 ± 0.002^a^	0.263 ± 0.025^bc^	0.229 ± 0.003^d^	0.242 ± 0.019^cd^	0.273 ± 0.002^b^
C12	0.433 ± 0.003^a^	0.362 ± 0.036^bc^	0.313 ± 0.007^d^	0.333 ± 0.028^cd^	0.372 ± 0.003^b^
C14	1.550 ± 0.008^a^	1.308 ± 0.131^bc^	1.128 ± 0.031^d^	1.197 ± 0.100^cd^	1.339 ± 0.006^b^
C15i	0.037 ± 0.001^a^	0.032 ± 0.003^b^	0.028 ± 0.000^c^	0.029 ± 0.003^bc^	0.032 ± 0.001^b^
C14:1 c9	0.131 ± 0.003^a^	0.110 ± 0.011^b^	0.096 ± 0.002^c^	0.102 ± 0.010^bc^	0.112 ± 0.001^b^
C15ai	0.081 ± 0.001^a^	0.069 ± 0.008^bc^	0.058 ± 0.002^d^	0.063 ± 0.006^cd^	0.072 ± 0.001^b^
C15	0.155 ± 0.001^a^	0.132 ± 0.012^bc^	0.113 ± 0.003^d^	0.121 ± 0.012^cd^	0.135 ± 0.001^b^
C16i	0.043 ± 0.000^a^	0.037 ± 0.004^b^	0.032 ± 0.003^c^	0.034 ± 0.004^bc^	0.037 ± 0.000^b^
C16	4.263 ± 0.026^a^	3.619 ± 0.357^bc^	3.125 ± 0.083^d^	3.314 ± 0.284^cd^	3.720 ± 0.016^b^
C16:1 c9	0.197 ± 0.003^a^	0.166 ± 0.017^bc^	0.143 ± 0.005^d^	0.154 ± 0.014^cd^	0.173 ± 0.002^b^
C17i	0.053 ± 0.001^a^	0.045 ± 0.004^bc^	0.038 ± 0.003^d^	0.041 ± 0.005^cd^	0.047 ± 0.001^b^
C17ai	0.089 ± 0.003^a^	0.076 ± 0.009^bc^	0.066 ± 0.003^d^	0.069 ± 0.007^cd^	0.080 ± 0.000^b^
C17	0.281 ± 0.008^a^	0.261 ± 0.023^abc^	0.252 ± 0.011^c^	0.254 ± 0.014^bc^	0.267 ± 0.003^b^
C17:1 c10	0.035 ± 0.002^a^	0.029 ± 0.003^bc^	0.024 ± 0.000^d^	0.028 ± 0.002^c^	0.031 ± 0.000^b^
C18	1.420 ± 0.009^a^	1.202 ± 0.121^bc^	1.038 ± 0.029^d^	1.102 ± 0.089^cd^	1.235 ± 0.002^b^
C18:1 t4	0.005 ± 0.000^a^	0.005 ± 0.001^a^	0.003 ± 0.000^b^	0.004 ± 0.001^ab^	0.005 ± 0.001^a^
C18:1 t5	0.007 ± 0.001^a^	0.005 ± 0.001^b^	0.006 ± 0.000^ab^	0.005 ± 0.001^b^	0.007 ± 0.001^a^
C18:1 t6-t9	0.076 ± 0.001^a^	0.070 ± 0.009^ab^	0.064 ± 0.003^b^	0.063 ± 0.005^b^	0.076 ± 0.001^a^
C18:1 t10	0.056 ± 0.001^a^	0.050 ± 0.007^bc^	0.045 ± 0.003^c^	0.046 ± 0.004^c^	0.053 ± 0.001^b^
C18:1 t11	0.213 ± 0.002^a^	0.181 ± 0.020^bc^	0.160 ± 0.003^d^	0.165 ± 0.014^cd^	0.188 ± 0.000^b^
C18:1 t12	0.053 ± 0.001^a^	0.045 ± 0.007^bc^	0.040 ± 0.002^c^	0.042 ± 0.002^c^	0.050 ± 0.001^b^
C18:1 c9	2.761 ± 0.021^a^	2.321 ± 0.233^c^	2.088 ± 0.060^c^	2.228 ± 0.193^c^	2.591 ± 0.001^b^
C18:1 t15	0.029 ± 0.001^a^	0.027 ± 0.005^ab^	0.026 ± 0.000^b^	0.025 ± 0.001^b^	0.029 ± 0.002^a^
C18:1 c11	0.082 ± 0.003^a^	0.068 ± 0.009^c^	0.064 ± 0.002^c^	0.067 ± 0.007^c^	0.078 ± 0.003^b^
C18:2 c9,c12	0.220 ± 0.001^c^	0.180 ± 0.019^d^	0.431 ± 0.015^b^	0.173 ± 0.011^d^	0.527 ± 0.003^a^
C18:2 c9,c15	0.025 ± 0.002^a^	0.020 ± 0.003^b^	0.018 ± 0.002^b^	0.019 ± 0.001^b^	0.020 ± 0.001^b^
C18:3 c6,c9,c12	0.008 ± 0.000	0.007 ± 0.001	0.007 ± 0.002	0.007 ± 0.001	0.007 ± 0.001
C18:3 c9,c12,c15	0.054 ± 0.002^c^	0.043 ± 0.005^d^	0.038 ± 0.001^d^	0.241 ± 0.017^b^	0.353 ± 0.006^a^
C18:2 c9,t11	0.083 ± 0.003^a^	0.062 ± 0.001^c^	0.073 ± 0.005^b^	0.067 ± 0.008^bc^	0.087 ± 0.005^a^
C18:2 t10,c12	0.004 ± 0.001^c^	0.004 ± 0.001^c^	0.005 ± 0.000^bc^	0.006 ± 0.001^b^	0.011 ± 0.002^a^
C18:2 CLA cc1	0.016 ± 0.001^a^	0.013 ± 0.002^bc^	0.011 ± 0.001^c^	0.012 ± 0.001^bc^	0.013 ± 0.000^b^
C18:2 CLA cc2	0.002 ± 0.000^b^	0.003 ± 0.000^a^	0.002 ± 0.000^b^	0.003 ± 0.001^ab^	0.003 ± 0.000^a^
C18:2 CLA t,t	0.013 ± 0.000^b^	0.010 ± 0.001^c^	0.014 ± 0.001^b^	0.012 ± 0.002^bc^	0.020 ± 0.000^a^
C18:3 c9,t11,c15	0.004 ± 0.000^c^	0.003 ± 0.000^d^	0.004 ± 0.001^cd^	0.106 ± 0.006^a^	0.054 ± 0.004^b^
C18:3 t9,t11,c15	0.002 ± 0.000^c^	0.003 ± 0.001^bc^	0.003 ± 0.000^b^	0.009 ± 0.004^a^	0.008 ± 0.000^a^
Σ CLA	0.117 ± 0.004^b^	0.091 ± 0.006^e^	0.105 ± 0.007^cd^	0.100 ± 0.012^d^	0.134 ± 0.006^a^
Σ CLNA	0.007 ± 0.001^c^	0.006 ± 0.001^c^	0.007 ± 0.001^c^	0.115 ± 0.009^a^	0.062 ± 0.004^b^
Σ SFA	9.022 ± 0.066^a^	7.652 ± 0.753^bc^	6.635 ± 0.180^d^	7.032 ± 0.585^cd^	7.867 ± 0.028^b^
Σ MUFA	3.645 ± 0.037^a^	3.076 ± 0.322^bc^	2.759 ± 0.081^c^	2.928 ± 0.250^c^	3.394 ± 0.007^b^
Σ PUFA	0.430 ± 0.003^c^	0.347 ± 0.035^d^	0.606 ± 0.024^b^	0.655 ± 0.052^b^	1.103 ± 0.020^a^
Σ FA	13.097 ± 0.106^a^	11.074 ± 1.110^c^	10.000 ± 0.286^c^	10.615 ± 0.887^c^	12.364 ± 0.002^b^

*Average value ± standard deviation (n = 3).

^a,b,c,d,e^Different superscript letters within a row for significant differences (p < 0.05).

c = *cis* double bound; t = *trans* double bound; ∑ = total; CLA = conjugated linoleic acid; CLNA = conjugated linolenic acid; SFA = saturated fatty acids; MUFA = monounsaturated fatty acids; PUFA = polyunsaturated fatty acids; FA = fatty acids.

ML: commercial skimmed milk, FCRL: fermented milk by *B. breve* NCIMB 702258; FS1: fermented milk with LA added; FS2: fermented milk with α-LNA added; FS3: fermented milk with LA and α-LNA added.

Commercial semi-skimmed milk fermentation over 24 h (FCRL) with *B. breve* NCIMB 702258 significantly decreased total FA content (11.074 ± 1.110 mg/mL vs. 13.097 ± 0.106 mg/mL in ML samples). All saturated (SFA), mono- (MUFA) and PUFA compounds were, in general, reduced especially in what concerns the total CLA content (0.091 ± 0.006 mg/mL vs. 0.117 ± 0.004 mg/mL).

Interestingly, this effect was even more pronounced in fermented samples with LA (FS1). Total FA content was 10.000 ± 0.286 mg/mL with values for main FA of 3.125 ± 0.083 mg C16/mL, 2.088 ± 0.060 mg C18:1 c9/mL and 1.038 ± 0.029 mg C18/mL. Regarding the results for CLA production, the concentration for this CFA was slightly lower (p < 0.05) than in ML samples (0.105 ± 0.007 mg/mL) while significantly higher than for FCRL, mainly in what concerns RA. Moreover, final LA concentration was 0.431 ± 0.015 mg/mL instead of the expected 0.680–0.720 mg/mL (LA naturally present in ML plus added LA) found in FCRL and ML samples.

In FS2 samples, utilization of α-LNA did not affect FA composition as compared to FCRL samples; there were no differences in total as well as main FA contents. Results were able to point out a significant synthesis of CLNA isomers (0.115 ± 0.009 mg/mL) mainly C18:3 c9,t11,c15 (0.106 ± 0.006 mg/mL), while final content for α-LNA, after 24 h, was 0.241 ± 0.017 mg/mL.

Finally, when both LA and α-LNA were added to ML, samples FMX showed a total FA content closest to that found in ML samples (12.364 ± 0.002 mg/mL; p < 0.05). SFA and MUFA levels were above those observed for FS1 and FS2 fermented milks. Moreover, FMX samples showed the highest PUFA content (p < 0.05) among all assayed samples. This can be associated to the observed amounts of LA (0.527 ± 0.003 mg/mL) and α-LNA (0.353 ± 0.006 mg/mL).

Interestingly, the presence of LA exerted an inhibitory effect on the α-LNA transformation into CLNA since in FMX samples total amount of CLNA was 0.062 ± 0.004 mg/mL in comparison to 0.115 ± 0.009 mg/mL found in FS2 samples (p < 0.05). Furthermore, CLA production (0.134 ± 0.006 mg/mL of total CLA) mainly RA and C18:2 CLA t,t, was also detected in higher amounts (p < 0.05) than in FS1.

Finally, it must be noted that CFA production during fermentation influenced the organoleptic quality of the final product; the CFA-enriched fermented milks presented a similar visual appearance to ML yet a less desirable aroma. From an applied perspective this observation reduces their organoleptic attractiveness to be used for dietary consumption (data not shown).

## Discussion

In previous studies, *B. breve* NCIMB 702258 revealed promising characteristics regarding CFA production^36^ and remodeling of host’s lipid composition^30^. However, few efforts have been carried out to find suitable food matrices to deliver this strain other than in a freeze-dried form used in animal experiments. Dairy products, mainly fermented products, are an ideal environment to assay elaboration of novel functional products based on microbiological production and activity. As previously reported and commented in this research work, *Bifidobacterium* sp. are able to produce CFA in reconstituted skim milk^27,37^, yet to the best of our knowledge commercial semi-skimmed milk (closer to technological reality) has not yet been studied. Furthermore, when comparing transformation rates between those studies using a defined medium used for microbiology purposes and those reported in this research work, it seems that conversion capacity is affected by matrix.

At the current moment there is a limited understanding about how these microorganisms transform PUFA into CFA. There are hypotheses suggesting that CFA production results from a detoxification mechanism^23,38^ to overcome the inhibitory potential of LA, α-LNA and other unsaturated fatty acids on bacterial growth and native fatty acid biosynthesis via the inhibition of enoyl-ACP reductase FabI^22^, the enzyme that controls the rate-limiting elongation step^20^. Under such rationale, *Butyrivibrio fibrisolvens* only started growth in the presence of LA after having reduced its initial concentration (50 µg/mL) up to 25%, converting this FA into RA and TVA^35^.

Therefore, this investigation hypothesized that PUFA exerting inhibitory effects on *B. breve* NCIMB 702258 must alter FA composition of membrane lipids, possibly palmitic and/or stearic acids.

Under this perspective it is important to first determine the highest tolerable concentration of unsaturated fatty acids that promotes no or little impairment of *B. breve* NCIMB 702258 growth since this tolerance will indicate further possibilities for production improvement. Although several authors have placed the limit at 0.5 mg LA/mL, it was hypothesized herein that the presence of a higher sub-lethal stress could lead the bacterial strain to produce higher amounts of CFA. Coakley *et al*.^33^ observed that optimum LA concentration for *B. breve* strains was 0.5 mg/mL after testing concentrations between 0.2 and 1.5 mg/mL. More recently, a research work, that assayed members of this genus, confirmed CFA synthesis in the presence of 0.5 mg substrate/mL^27^ but also showed that LNA exerted a stronger inhibitory effect on bacterial growth than LA^27^.

In this current research work, results confirmed that utilization of 1 mg/mL of LA was deleterious for the *B. breve* strain as reflected in the less positive growth performance based on OD_600_ measurements. Furthermore, concentrations of 2 mg/mL of LA clearly exerted lethal injury although preliminary assays in agar plates suggested that this *B. breve* strain supported such a high concentration. Indeed, in previous experiments with *B. breve* KCTC 3461, this bacterium was able to tolerate 5 mg/mL of LA, after previous adaptation in culture broth with 0.5 mg/mL^39^. However, in this study, even low concentrations of LA had negative effects on *B. breve* NCIMB 702258 and it was not able to grow at 5 mg/mL. These differences may be attributed to specific strain characteristics and sustain LA/LNA tolerance as a strain-specific trait.

Interestingly, the presence of precursor substrate LA, α-LNA and γ-LNA did not affect *B. breve* NCIMB 702258 viable cell numbers to the extent that the type of matrix – cys-MRS medium versus commercial semi-skimmed milk (ML) - did, which highlights the importance of the growth medium. While in cys-MRS, the addition of the different assayed FA lowered viable cell numbers in relation to the control, such effects were not found when assaying the same variables in ML; indeed, viable cell numbers were similar independent of the absence or presence of an added FA. *Bifidobacterium breve* NCIMB 702258 was first isolated from the infant gut^40^ which may justify its good tolerance to dairy environments. Elsewhere it was also concluded that milk proteins exert protective effects on bacteria stability and viability^41^.

In previous research works, when inoculated in culture medium *B. breve* NCIMB 702258 was able to convert up to 65% LA into CLA when grown in the presence of 0.5 mg LA/mL or 59.7% of LA and 79.1% of α-LNA into their corresponding CFA, when grown in the presence of 0.4 mg LA/mL^37,40^. These observations show that slight variations in the tested conditions can result in variable conversion rates, a trend further demonstrated by Hennessy *et al*.^36^ when, in the presence of 0.45 mg/mL, 60.0% and 49.7% of LA and α-LNA were bioconverted, respectively.

The results obtained in the present study align partially with these observations, since when PUFAs were added separately, CLNA production capacity was similar to the abovementioned results yet CLA production capacity was lower. The use of a slightly lower LA concentration or longer incubation period by the abovementioned studies could be the reason for such difference. However, it must be highlighted that reported data showed that this *Bifidobacterium* strain reaches the stationary phase in less than 12 h.

Furthermore, although Hennessy *et al*.^36^ showed that *B. breve* NCIMB 702258 could convert γ-LNA (37.5%), in the present research work the production of any possible conjugated isomers was not detected in the presence of this substrate. Accordingly, results reported herein point out that LA and α-LNA were reduced to a higher extent than γ-LNA and were also highly accumulated in the membrane lipids. This seems to suggest that CFA production is associated with absorption of precursor substrates by the membrane and indeed, some linoleate isomerases from *Propionibacterium* and *Lactobacillus* have been described as membrane proteins^42,43^.

An important observation reported in this study is that *B. breve* NCIMB 702258 was able to isomerize α-LNA more efficiently than LA, independently of the substrate being added alone or in combination. There are two possible explanations for this fact. The first is related to the toxicity and stress onset capacity of the added FA, where if α-LNA is more toxic than LA then the abovementioned detoxification mechanism of biohydrogenation may be more intensively activated^44^. The same hypothesis has been proposed to explain the capacity of probiotic strains to convert LA into CLA isomers^45^ and assays with ruminal bacteria revealed that LNA was more toxic than LA^46^. The second explanation upholds the possibility that linoleate isomerase (LAI) has a higher specificity for α-LNA than LA – LAI is described as being the enzyme responsible for LA and LNA conversion^47^.

Analysis of FA composition of pellets from *B. breve* NCIMB 702258, revealed an accumulation pattern (LA > γ-LNA > α-LNA) when these FA were added either separately or together. As most linoleate isomerase enzymes (LAI) (i.e. the proteins involved in such transformations) are membrane associated^24,48^, it can be hypothesized that the substrate enters into the membrane to be subsequently isomerized and then released as CFA into the medium. This hypothesis stated herein is based on the fact that as CLA and CLNA contents increased among the membrane lipids when LA and/or α-LNA were tested.

Moreover, stearic and oleic acids decreased in the pellets of assayed precursors of CFA, especially with γ-LNA, a substrate which the strain could not convert into conjugated forms. This may suggest that CFA production is regulated by the FA synthesis pathway.

Cyclopropane FA in bacteria are synthesized from cis unsaturated FA attached to phospholipids^49^, such as conversion of cis vaccenic acid (C18:1 c11) into lactobacillic acid (cyc C19) in wine malolactic fermentation by lactic acid bacteria^50,51^. Intriguingly, utilization of γ-LNA in the growth medium led to the lowest concentration of cis vaccenic acid. Therefore, it cannot be excluded that phospholipids synthesis and metabolism play a role in the action of LAI or other enzymes involved.

Some authors have reported that gut microbiota as well as strains from the genera *Streptococcus* and *Lactobacillus* produced hydroxy and oxo fatty acids from LA and LNA, suggesting that those FA are intermediate products in the CLA and CLNA synthesis metabolic routes^24,38,52^. However, such compounds were not detected with *B. breve* NCIMB 702258.

Besides fundamental issues, a major objective of this study was to assess the possibility of using *B. breve* NCIMB 702258 for the manufacture of a potentially functional novel dairy product according to the previously reported health effects for CLA, CLNA as well as for the strain itself. Although, it was possible to obtain CFA-enriched fermented products, quantified amounts of CFA were lower than in cys-MRS culture medium. Interestingly, this strain grew better in commercial semi-skimmed milk even in the presence of the assayed substrates, an important asset if its probiotic potential is sought. All such trends, confirm the idea that CLA and CLNA production capacities are associated to mechanisms to avoid deleterious effects by stress agents.

As commented above this strain has been previously assayed in skimmed milk but commercial fermented milks normally have fat contents of 1.6–2% fat. Thus, the matrix may affect the conversion efficiency. All the assayed products showed that fermentation resulted in lower contents of SFA and MUFA. A previous research work assaying stability of fermented products elaborated with milk enriched in CLA, found similar effects which were directly associated to microbial growth^53^. When comparing kefir elaborated with grains from different geographical areas in Brazil, some samples have lower SFA contents due to the action of microbial Δ9 desaturase activity^54^.

In the European market, the serving size of commercial fermented milks ranges from 100 to 300 mL. According to the CFA contents in the proposed product, intake would result in 10.5–31.5 mg CLA for FS1, 11.5–34.5 mg CLNA for FS2 and 13.4–40.3 mg CLA/6.2–18.6 mg CLNA for FMX. As discussed in the introduction section, the effective dose for CLA in humans is 3–6 g/day^7^ while for CLNA is 2–3 g/day^8^. Thus, at least in the current assayed conditions, fermented dairy products manufactured using *B. breve* NCIMB 702258 do not provide the proposed amount needed to exert the biological health effects of CLA and CLNA. However, some studies have concluded that this strain can alter the lipid composition of the host^29,30,55^ opening further promising alternatives for utilization of its health benefits. Further studies may be focused on studying the positive health effects of fermented dairy products using *B. breve* NCIMB 702258.

## Conclusion

The current research has shown that the CFA production by *B. breve* 702258 is closely associated to stress adaptation mechanisms that may be regulated by the FA and phospholipids metabolisms. On the other hand, although proposed fermented products do not provide the required bioactive dose of CFA through common serving size, it has been demonstrated that aside of organoleptic characteristics that have to be improved in a future, semi-skimmed dairy products are a suitable matrix for delivery of the potentially bioactive *B. breve* NCIMB 702258, an important novel highlight of this study.

## Materials and Methods

### Analytical reagents

Hexane, methanol, dimethylformamide (DMF) and acetonitrile (AcN) were HPLC grade (VWR Chemicals, West Chester, PA). GLC-Nestlé36 FAME mix and glyceryl tritridecanoate (99.9%) were obtained from Nu-Chek Prep, inc. (Elysian, Minnesota, USA). Undecanoic acid (99.9%) and N,O-Bis(trimethylsilyl)trifluoroacetamide (BSTFA) were acquired from Alfa Caesar (Haverhill, MA, USA) and butterfat CRM-164 (EU Commission; Brussels, Belgium) from Fedelco Inc. (Madrid, Spain). Sulphuric acid was obtained from Fisher Scientific (Hampton, NH, USA), while sodium methoxide was from Acros Organics (Geel, Belgium). Supelco 37 FAME mix, bacterial FAME (BAME mix), linoleic acid (LA; C18:2 c9,c12), α-linolenic acid (α-LNA; C18:3 c9,c12,c15) and γ-linolenic acid (γ-LNA; C18:3 c6,c9,c12) were purchased from Sigma-Aldrich (St. Louis, MO, USA).

### Culture conditions

*Bifidobacterium breve* NCIMB 702258 (NCIMB, Aberdeen, Scotland), stored at −80 °C in glycerol 30% (w/w) (Fisher Scientific), was activated at 2% (v/v) in MRS broth (Biokar Diagnostics, Beauvais, France) supplemented with 0.05% (w/v) L-cysteine-HCl (Sigma-Aldrich, St. Louis, MO, USA) and incubated overnight at 37 °C in an anaerobic workstation (Whitley DG 250; Don Withley Scientific, Yorkshire, UK) under a mixture of 80% nitrogen, 10% hydrogen and 10% carbon dioxide. About 10% (v/v) of the activated culture was then transferred to fresh cys-MRS medium (pre-inoculation) and incubated at 37 °C for 16 h under anaerobic conditions. Afterwards, 2% (v/v) of the pre-inoculum was spiked into new medium (10 mL) for the following experiments.

### Determination of maximum tolerance to octadecenoic FA

In order to know the substrate tolerance by *B. breve* NCIMB 702258, tests were conducted using LA according to the conditions previously described^56^ with slight modifications. Briefly, after activation, the strain was inoculated in cys-MRS agar (Biokar Diagnostics) plates containing 1, 2 or 5 mg/mL (i.e 0.1, 0.2 and 0.5% w/v) of LA, added from a stock solution at 50 mg/mL with 2% (w/v) Tween 80 (Sigma-Aldrich), and incubated for 48 h at 37 °C. Plates without substrate were used as control.

*B. breve* NCIMB 702258 from plates containing 0, 1 and 2 mg LA/mL were subsequently used to spike well plates containing 200 µL of MRS and the same LA concentration than in the corresponding agar plate except for 0 (control plates) that was assayed at 0.5 mg LA/mL. For each concentration, absence of substrate was also tested. Each well was covered with 50 µL paraffin to ensure anaerobic environment. Growth was monitored in plate reader (model FLUOSTAR optima; BMG labtech, Ortenberg, Germany) at 600 nm during 48 h at 37 °C. All experiments were carried out, at least, in duplicate.

### CLA and CLNA production in culture medium

Stock solutions of LA and α-LNA were prepared at 15 mg/mL and of γ-LNA at 10 mg/mL with 2% (w/v) Tween 80 and homogenized by means of an Ultra-Turrax (IKA Works, Inc., Wilmington, NC, USA) using 15000 rpm during 150 s (3 intervals separated 30 s), before filter-sterilization through a 0.20 μm-pore size membrane (Millipore, Burlington, MA, USA). These were stored at 4 °C until further use. *Bifidobacterium breve* NCIMB 702258 was inoculated at 2% (v/v) in new cys-MRS broth (10 mL) without any substrate (control; CRL) or containing LA (S1), α-LNA (S2) or γ-LNA (S3) added separately or together (MIX) to a final concentration of 0.5 mg/mL each FA. Samples were incubated at 37 °C for 24 h under anaerobic conditions. All experiments were carried out in triplicate. Bacterial growth was analyzed through plating on cys-MRS agar plates of sequential decimal dilutions followed by viable cell numbers determination. The samples were collected after centrifugation at 1250 g, 18 °C for 5 min. Afterwards, supernatant and pellet were both assayed for CLA and CLNA quantification.

### CLA and CLNA production in semi-skimmed milk

*Bifidobacterium breve* NCIMB 702258 was inoculated at 2% (v/v) in a commercial UHT semi-skimmed milk (1.6% fat; ML) (10 mL), purchased at a local supermarket, containing 0.05% (w/v) of L-cysteine-HCl and no precursor substrate (control; FCRL) or LA (FS1), α-LNA (FS2), or both (FMX) to a final concentration of 0.5 mg/mL each FA. Samples were incubated at 37 °C for 24 h in an anaerobic workstation as previously described. All experiments were carried out in duplicate. Bacterial growth was analyzed through plating of sequential decimal dilutions followed by viable cell numbers determination. CLA and CLNA contents were measured according to the following procedures:

#### Fatty acids analysis

For the fatty acids (FA) analysis, stock solutions, supernatants and fermented milk samples (500 μL) or pellets (500 mg) were prepared according to Pimentel *et al*.^57^. Briefly, for quantification purposes, samples were added with 100 µL of tritridecanoin (1.34 mg/mL) and undecanoic acid (1.5 mg/mL) prior to derivatization. Then 2.26 mL of methanol were added, followed by 1 mL of hexane and 240 µL of sodium methoxide (5 M). Samples were vortexed and incubated at 80 °C for 10 min. After cooling in ice, 1.25 mL of DMF were added prior to 1.25 mL of sulphuric acid (3 M; prepared daily). Samples were vortexed and incubated at 60 °C for 30 min. Finally, after cooling, 1 mL of hexane was added, and samples were vortexed and centrifuged (1250 g; 18 °C; 5 min.). The upper layer containing methyl esters (FAME) was collected for further analysis.

#### Analysis of hydroxy-FA

For the analysis of hydroxyl fatty acids as trimethylsilyl (TMS) derivatives in pellet samples, after obtaining the FAME, solvent was evaporated in a stream of nitrogen. Then it was added 150 µL of BSTFA and 500 µL of AcN followed by incubation at 70 °C for 30 min^58^. Finally, AcN was evaporated in a nitrogen stream and resuspended in 500 µL of hexane. Samples before and after silylation were analysed.

#### Gas Chromatography conditions

FAME and TMS were both analyzed in a gas chromatrograph HP6890A (Hewlett-Packard, Avondale, PA, USA), equipped with a flame-ionization detector (GLC-FID) and a BPX70 capillary column (60 m × 0.32 mm × 0.25 μm; SGE Europe Ltd, Courtaboeuf, France). Analysis conditions were as follows: injector temperature 250 °C, split 25:1, injection volume 1 μL; detector (FID) temperature 275 °C; hydrogen was carrier gas at 20.5 psi; oven temperature program: started at 60 °C (held 5 min), then raised at 15 °C/min to 165 °C (held 1 min) and finally at 2 °C/min to 225 °C (held 2 min). Supelco 37, FAME from CRM-164 and BAME mix were used for identification of fatty acids. GLC-Nestlé36 was assayed for calculation of response factors and detection and quantification limits (LOD: 0.79 ng FA/mL; LOQ: 2.64 ng FA/mL).

### Statistical analysis

Results are reported as mean values ± standard deviation. Data were first analyzed for normality distribution. Levene’s test was applied to verify the homogeneity of the variances. Afterwards, t-Student test was applied when comparing means of two groups and one-way ANOVA for three or more groups. Tukey post hoc test was used to determine differences within groups. Level of significance was set in general at 0.05; for growth experiments CFU differences had to be above 2 log. Analyses were performed using the IBM SPSS Statistics 24 (SPSS Inc., IBM Corporation, NY, USA).
