# Publisher Correction: Effect of Pufa Substrates on Fatty Acid Profile of *Bifidobacterium breve* Ncimb 702258 and CLA/CLNA Production in Commercial Semi-Skimmed Milk

**DOI:** 10.1038/s41598-019-40320-3

**Published:** 2019-03-12

**Authors:** Ana Luiza Fontes, Lígia Pimentel, Luis Miguel Rodríguez-Alcalá, Ana Gomes

**Affiliations:** 1 Universidade Católica Portuguesa, CBQF - Centro de Biotecnologia e Química Fina – Laboratório Associado, Escola Superior de Biotecnologia, Porto, 4200-374 Portugal; 2 CINTESIS – Centro de Investigação em Tecnologias e Sistemas de Informação em Saúde, Faculdade de Medicina da Universidade do Porto, Porto, 4200-450 Portugal; 3 QOPNA – Unidade de Investigação de Química Orgânica, Produtos Naturais e Agroalimentares, Universidade de Aveiro, Campus Universitário de Santiago, Aveiro, 3810-193 Portugal; 4 Centro de Investigación en Recursos Naturales y Sustentabilidad (CIRENYS), Universidad Bernardo O’Higgins, Santiago, de Chile Chile

Correction to: *Scientific Reports* 10.1038/s41598-018-33970-2, published online 22 October 2018

The Author Contributions section in this Article contains an error.

“LR, LP and AG designed the experiments, upon hypotheses conceived by LR. A.F. conducted the experimental work under supervision of L.R., L.P. and A.G. A.F., L.R. and L.P. performed statistical analysis. L.R. and L.P. accomplished data interpretation. A.F. and L.R. wrote the manuscript. L.R. and A.G. contributed to the critical review of the manuscript responsibility for the final content of the manuscript. All authors read and approved the final manuscript. The work is part of a financed project led by L.R.”

should read:

“LR, LP and AG designed the experiments, upon hypotheses conceived by LR. A.F. conducted the experimental work under supervision of L.R., L.P. and A.G. A.F., L.R. and L.P. performed statistical analysis. L.R. and L.P. accomplished data interpretation. A.F. and L.R. wrote the manuscript. L.R. and A.G. contributed to the critical review of the manuscript. All authors read and approved the final manuscript. The work is part of a financed project led by L.R.”

